# The Effects of Human Caring Theory-Based Interventions on Women’s Mental Health: A Systematic Review

**DOI:** 10.3390/healthcare14121658

**Published:** 2026-06-11

**Authors:** Şehma Şen, Şeyma Demiralay

**Affiliations:** 1 Nursing (English) Department, İstanbul Atlas University, Anadolu Street, İstanbul 34408, Türkiye; 2 Nursing Department, İstanbul Atlas University, Anadolu Street, İstanbul 34408, Türkiye; seyma.demiralay@atlas.edu.tr

**Keywords:** mental health, nursing, psychological well-being, Watson’s Theory of Human Caring, women’s health

## Abstract

**Highlights:**

**What are the main findings?**

- Nursing interventions based on Watson’s Theory of Human Caring significantly reduce anxiety, depression, and stress levels among women facing traumatic health events such as pregnancy loss, infertility, and cancer.
- The application of Caritas processes effectively bolsters internal psychological resources, leading to measurable increases in self-efficacy, hope, and adaptive coping mechanisms across various clinical samples in the Middle Eastern region.

**What are the implications of the main findings?**

- Integrating Watson’s holistic framework into routine clinical protocols provides a standardized yet humanistic approach to protecting women’s mental health during critical life transitions.
- The findings encourage nursing educators and healthcare administrators to embed transpersonal caring principles into curricula and hospital environments to foster resilience in both patients and care providers.

**Abstract:**

**Background/Objectives**: This systematic review aims to synthesize existing evidence on the impact of nursing interventions based on Watson’s Theory of Human Caring (THC) on women’s mental health and to provide an evidence-based framework for clinical practice. **Methods**: The review followed the PRISMA guidelines and was registered in the PROSPERO database (Registration No: CRD420251111577). A comprehensive literature search was conducted across databases, including PubMed, CINAHL, and Google Scholar. Ten studies (nine randomized controlled trials and one quasi-experimental study), involving 869 participants, met the eligibility criteria. Data were analyzed using a narrative synthesis approach due to methodological and clinical heterogeneity. **Results**: A total of 10 studies involving 894 women met the inclusion criteria. Geographically, nine studies were conducted in Türkiye and one in Iran. The included studies spanned various clinical contexts directly associated with significant mental health challenges for women, including medical abortion, infertility, gynecological oncology, and the postpartum period. The synthesized findings demonstrated that nursing interventions based on Watson’s Human Caring Theory led to statistically significant reductions in anxiety, depression, stress, postpartum depression risk, and infertility-related distress. Furthermore, these caritas-based frameworks significantly enhanced positive psychological assets, including self-efficacy, hope, meaning in life, prenatal attachment, and social support perception. **Conclusions**: Watson’s Theory of Human Caring provides a transformative framework for women’s health nursing that extends beyond symptom management to strengthen the individual’s internal resources and spiritual integrity. Integrating this theory into clinical protocols and nursing curricula is essential for humanizing care and protecting women’s mental health during challenging life transitions, particularly within the examined sociocultural contexts.

## 1. Introduction

Developed by Jean Watson in the late 1970s during her work at the University of Colorado, Watson’s Theory of Human Caring (THC) provides a comprehensive theoretical framework aimed at introducing a holistic, ethical, and humanistic perspective to nursing care [1]. The theory centers on a holistic state of well-being that encompasses not only physical dimensions but also emotional, mental, social, and spiritual aspects of the individual [2]. In this context, the caring process is defined as an interpersonal, authentic, and meaning-centered relationship.

In addition to offering a philosophical and ethical foundation for nursing practice, THC supports an interdisciplinary approach that integrates the scientific and humanistic dimensions of care [3]. The caritas processes that constitute the core of the theory include elements such as love, compassion, hope, trust, empathic listening, expression of emotions, problem solving, authentic teaching–learning, and the creation of a healing environment [2,4]. By emphasizing the unity of mind, body, and spirit and highlighting the caring dimension of medicine and nursing, the theory embraces new dimensions of care and their intersections, aiming to transform the caring process into a field of transformative interaction [4,5].

THC reflects the essence of nursing and incorporates concepts such as love, compassion, self-compassion, respect, trust, human dignity, morality, and ethics [2]. It includes universal principles of caring and can be applied to individuals from diverse religious, linguistic, cultural, and belief backgrounds [4]. The interaction between nurse and patient is not limited to physical contact but also involves deepening through existential integration [2]. THC values not only the care provided to the individual but also the presence and well-being of the caring nurse. In this respect, it encourages a reciprocal developmental process that supports the well-being of both care recipients and caregivers [4].

The theory provides guidance for nursing practice and contributes to the positive development of care outcomes [6,7,8]. For this reason, it has been widely used in nursing practice and research with different samples for many years [6,7,8]. One of the areas in which the theory has been applied is women’s mental health [9,10].

Throughout the life cycle, women may encounter experiences that generate intense biopsychosocial stress, such as pregnancy, childbirth, infertility, motherhood, menopause, chronic illnesses, and cancer [11,12,13]. In addition, due to social roles, caregiving burden, economic inequalities, and social expectations, women may occupy a more vulnerable position in terms of mental health [14]. This situation increases the prevalence of anxiety, depression, stress, and adjustment problems among women and strengthens the need for holistic care approaches that support mental health [13,15,16]. In this context, theory-based interventions that focus not only on symptoms but also on individuals’ meaning-making processes and lived experiences hold significant potential for the protection and promotion of women’s mental health [9,10,17,18,19].

In recent years, an increase has been observed in the number of randomized controlled and quasi-experimental studies employing THC-based nursing interventions in female samples [9,10,20]. These studies have demonstrated the positive effects of THC-based care on women’s psychological well-being in various fields, including infertility, gynecological cancer, breastfeeding education, and psychological adjustment. However, the findings of existing studies have been obtained in different contexts, using different methods, and with limited samples. Therefore, there is a need for a comprehensive and up-to-date systematic review that synthesizes these results from a holistic perspective.

Although systematic reviews addressing the effects of nursing theory-based interventions in general patient populations are available in the literature [21,22], no study has been identified that systematically evaluates experimental and quasi-experimental studies focusing specifically on the effects of THC on women’s mental health. This gap constitutes an important limitation in strengthening evidence-based decision-making processes in nursing practice.

The aim of this systematic review is to systematically evaluate the findings of experimental and quasi-experimental studies examining the effects of Watson’s Theory of Human Caring-based interventions on women’s mental health, to present the effects of these interventions on variables such as psychological well-being, anxiety, depression, stress, and adjustment from a holistic perspective, and to develop evidence-based recommendations for nursing practice. It is expected that this study will contribute to the dissemination of THC-based care approaches in the field of women’s mental health, strengthen clinical practices, and guide future research.

## 2. Materials and Methods

### 2.1. Protocol and Registration

Focusing on experimental trials, this study utilizes a systematic review design conducted in accordance with the PRISMA reporting standards. The review protocol was officially registered in the PROSPERO database (Registration No: CRD420261333520) in July 2025 to ensure procedural clarity. To ensure objectivity, the literature search, selection, and data extraction phases were performed independently by two researchers (S.S., S.D.K.), with final decisions finalized during consensus meetings with the entire authorship team. Additionally, a pilot phase was executed with the participation of both authors to test the effectiveness of PubMed search strings and assessment protocols, ensuring a robust and reliable research framework.

### 2.2. Eligibility Criteria

The PICOS (Participants, Interventions, Comparisons, Outcomes, Study Design) framework was used to define the scope of the research question [23]. This review focused on experimental and quasi-experimental studies investigating the effects of nursing interventions based on Watson’s Theory of Human Caring (THC) on women’s mental health. The effectiveness of these interventions was evaluated against standard care by measuring outcomes such as depression, anxiety, stress, hope, and self-efficacy. Studies published in English or Turkish without date restrictions were deemed eligible. Conversely, observational studies, animal experiments, publications with unclear methodology, and research based on models other than THC were excluded. Furthermore, studies with unclear methodology—specifically defined as those lacking explicit descriptions of their research design, intervention protocols, or statistical analysis methods—were excluded from the review.

### 2.3. Search Strategy

A comprehensive search of electronic databases was undertaken between January 28 and February 9, 2026, to identify relevant literature. The databases queried were PubMed, EBSCO (Medline, CINAHL), Embase (OVID), Web of Science, PsycINFO (via Ovid SP), Scopus, and Cochrane. In addition to the database search, the bibliographies of all eligible studies were manually screened to identify any other potentially relevant publications.

The keywords used in the review of international databases included (“Women” OR “Woman” OR “Female”) AND (“Nursing Theory” OR “Human Caring Theory” OR “Theory of Human Caring” OR “Watson’s Theory” OR “Watson’s Model” OR “Caritas Process” OR “Caring Science”) AND (“Mental Health” OR “Well-being” OR “Psychological well-being” OR “Anxiety” OR “Depression” OR “Stress” OR “Emotional states”). After finding the eligible studies, their reference lists were checked to determine the need for further search.

### 2.4. Study Selection

The systematic management and screening of identified records were facilitated through specialized software platforms. Initially, all retrieved citations were exported to EndNote 20 (Clarivate, Philadelphia, PA, USA), where duplicate entries were identified and removed to ensure a unique dataset. The subsequent screening process was conducted independently by two reviewers using the Covidence platform (Veritas Health Innovation, Melbourne, Australia; https://www.covidence.org), encompassing a rigorous evaluation of titles, abstracts, and full-text versions. Adherence to the pre-established inclusion and exclusion criteria was maintained throughout each phase; any discrepancies between reviewers were resolved through collaborative discussion until a consensus was reached. To maintain systematic accuracy and data integrity, a standardized extraction form was employed for data collection. The comprehensive study selection process is illustrated in the PRISMA flow diagram.

### 2.5. Data Extraction

Systematic data extraction was executed for each included study using a rigorously developed standardized template. This form was strategically structured to capture essential variables, including bibliographic details (author, year), methodological characteristics (study design, setting, objective, sample size), participant demographics (intervention and control groups), and the specifics of nursing interventions based on Watson’s Theory. Additionally, primary and secondary outcomes, as reported in the original studies, were meticulously recorded. To enhance reliability and minimize potential bias, the extraction process was performed independently and in duplicate by the first and second authors. Any discrepancies identified during this phase were resolved through a final joint verification and consensus-building step, ensuring the integrity and accuracy of the synthesized data (Table 1).

### 2.6. Methodological Quality Assessment of Studies

To evaluate the methodological rigor and risk of bias, the Joanna Briggs Institute (JBI) Critical Appraisal Tools were utilized. Depending on the specific design of the included studies, either the 13-item checklist for Randomized Controlled Trials or the 9-item checklist for Quasi-Experimental Studies was applied [31]. The appraisal process was initially conducted independently by two reviewers (the first and second authors) to ensure objectivity. Any discrepancies in the scoring were reconciled through a consensus meeting with a third senior researcher, leading to a finalized assessment for each study. Following the framework by Goldsmith et al., the final quality scores were calculated and categorized as “good” (80–100%), “fair” (50–79%), or “low” (<50%) quality [32]. This systematic classification allowed for a clear determination of the evidentiary weight of each study within the review.

### 2.7. Data Analysis

A narrative synthesis approach was employed to integrate and summarize the findings from the included studies. Due to significant methodological and clinical heterogeneity—specifically the diversity in participant populations (e.g., medical abortion, infertility, oncology, and postpartum groups) and the varying durations and formats of interventions based on Watson’s Theory of Human Caring—a formal meta-analysis was deemed inappropriate.

The synthesis process followed a systematic three-stage path. First, data concerning study characteristics, participant demographics, intervention protocols, and quality appraisal results were tabulated to provide a comprehensive structural overview. Second, the findings were categorized according to primary outcomes, including anxiety, depression, stress, self-efficacy, and quality of life. Third, within these thematic clusters, patterns of effectiveness and consistencies in clinical outcomes were analyzed. Any contradictory evidence was interpreted descriptively to draw meaningful conclusions regarding the overall impact and applicability of Watson’s model-based interventions.

## 3. Results

### 3.1. Search Results

**Figure 1:**
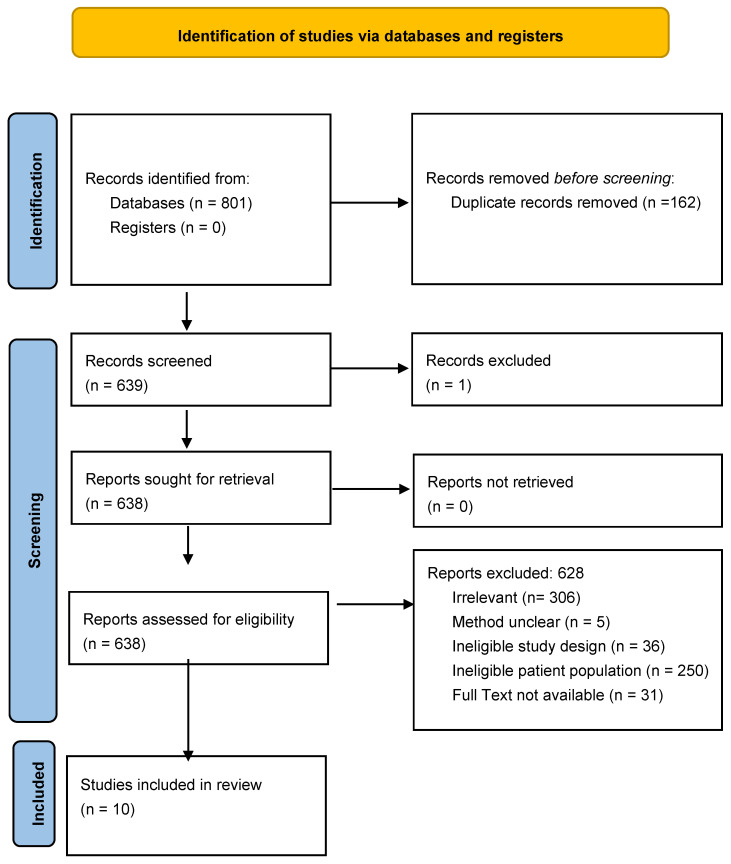
PRISMA flow diagram.

A total of 801 records were initially identified through a comprehensive search of electronic databases. Following the removal of 162 duplicate entries via Covidence and manual checking, the remaining 639 records underwent a rigorous screening of titles and abstracts. This stage resulted in the selection of 11 potential articles for a more detailed full-text assessment. During the full-text review, one study was excluded because its sample did not meet the predefined eligibility criteria. Consequently, 10 studies that strictly adhered to the inclusion criteria were included in this systematic review. The entire study selection process and the reasons for exclusion are detailed in the PRISMA flow diagram (Figure 1).

### 3.2. Characteristics of Studies

Of the ten studies included in this systematic review, nine were designed as randomized controlled trials, and one was a quasi-experimental study. All included research was published between 2013 and 2025 and was conducted exclusively in Türkiye. The total sample size across these studies was 869 participants, comprising 437 in the intervention groups and 432 in the control groups. The participants were adult women from diverse clinical backgrounds, including those who underwent medical abortion, received infertility treatment, were in the pregnant or postpartum period, experienced pregnancy loss, or were diagnosed with gynecological cancer. Across all studies, nursing interventions were developed and implemented based on Watson’s Theory of Human Caring.

### 3.3. Characteristics of Participants

The total sample size across the ten included studies was 869, with individual study populations ranging from 60 to 102 participants. The research focused on diverse clinical populations within women’s health, all of whom received care or education based on Watson’s Theory of Human Caring. These populations can be categorized into four main clinical groups.

#### 3.3.1. Women in the Pregnancy and Postpartum Periods

A significant portion of the studies focused on the transition to motherhood and the postpartum period. This group included pregnant women receiving psycho-educational interventions to prevent postpartum depression and enhance breastfeeding self-efficacy. Additionally, women in the healthy postpartum period were studied to evaluate the impact of human caring-based education on maternal well-being and neonatal care [10,27,28].

#### 3.3.2. Women Experiencing Pregnancy Loss or Medical Abortion

Research in this category addressed the psychological needs of women following the termination of pregnancy. This included women who underwent medical abortion and those who became pregnant again following a previous pregnancy loss, focusing on reducing levels of anxiety, depression, and stress through caritas-based nursing care [20,29].

#### 3.3.3. Women Undergoing Infertility Treatment

Several studies targeted women facing the challenges of infertility. These samples comprised women undergoing active infertility treatments and those experiencing treatment failure. The interventions aimed to improve adjustment, increase perceived self-efficacy, and manage the distress associated with the infertility process [24,25].

#### 3.3.4. Women with Gynecological Cancer

This group included women diagnosed with gynecological cancers undergoing clinical treatment. The research focused on the effects of holistic nursing interventions, such as reflexology integrated with Watson’s model, to manage symptoms of anxiety and depression and to improve the overall quality of life during the oncological process [9,26,30].

### 3.4. Characteristics of Interventions

The interventions in the studies included in this review were systematically grounded in Watson’s Theory of Human Caring (THC) and the 10 Caritas Processes, although they exhibited variations in terms of delivery methods, duration, and specific components. In several studies, THC was implemented as a structured, face-to-face psycho-educational or counseling program to manage outcomes such as postpartum depression, breastfeeding self-efficacy, or distress related to infertility. These programs were typically delivered over multiple sessions, ranging from three to six weeks, and often incorporated clinical education alongside caritas-based therapeutic communication.

In some studies, Watson’s model served as the theoretical framework for integrated, multi-modal interventions. For instance, the caritas processes were combined with physical relaxation techniques, most notably foot reflexology, to alleviate anxiety and depression while improving the quality of life in women with gynecological cancers. Other research utilized THC to develop comprehensive “Clinical Care Programs” or “Home-Based Care Models,” specifically designed to provide continuous support for women following medical abortion or pregnancy loss.

To enhance accessibility and continuity of care, several interventions adopted a blended delivery approach. These included a combination of face-to-face hospital sessions with asynchronous distance education modules, telephone counseling, and support via mobile applications (e.g., WhatsApp). These customized support systems were particularly prevalent in studies focusing on the prenatal and postpartum periods. While many interventions were compared against standard routine care, some studies also evaluated the effectiveness of THC-based programs against traditional hospital protocols to assess their impact on self-efficacy, psychological adjustment, and stress management across various women’s health contexts.

### 3.5. Quality Assessment Results

Following the assessment, it was observed that the quality scores of the randomized controlled trials (*n* = 8) ranged from 69.2% to 76.9%. The most frequently identified methodological shortcomings in these studies were the lack of blinding of participants and practitioners, unclear allocation concealment, and, in some instances, the absence of assessor blinding. Additionally, while most trials maintained high follow-up rates, the intention-to-treat principle was not clearly applied in all analyses. Conversely, the two quasi-experimental studies evaluated were found to possess high methodological quality, meeting the relevant criteria on the JBI checklist. Despite the variations in methodological scoring, all evaluated studies were categorized as having “fair” to “good” quality and were deemed to be of sufficient quality for inclusion in this review (Table 2).

### 3.6. Data Collection Tools

In the studies included in this review, a variety of validated measurement tools were used to assess the multifaceted effects of nursing interventions based on Watson’s Theory of Human Caring. Among the psychological variables, anxiety and depression were the most frequently examined outcomes. Anxiety was predominantly measured using the Beck Anxiety Inventory (BAI) and the State-Trait Anxiety Inventory (STAI), while depression levels were most assessed with the Beck Depression Inventory (BDI) and the Edinburgh Postpartum Depression Scale (EPDS), particularly in studies involving pregnant and postpartum women.

To determine general stress and distress levels, the Perceived Stress Scale (PSS) and the Infertility Distress Scale (IDS) were frequently preferred. Self-efficacy emerged as another significant outcome variable, measured through the Breastfeeding Self-Efficacy Scale (BSES) and the Self-Efficacy-Expectancy Scale. Furthermore, the Visual Analog Scale (VAS) was utilized in clinical contexts to evaluate subjective experiences such as pain. Quality of life, especially in gynecological cancer populations, was examined using the European Organization for Research and Treatment of Cancer Quality of Life Questionnaire (EORTC QLQ-C30). In addition to these, more specific scales were employed, such as the Prenatal Attachment Inventory (PAI) to assess maternal-fetal bonding and the Postpartum Adjustment Questionnaire to evaluate psychological adaptation after childbirth.

### 3.7. Outcomes

An analysis of the outcomes from the included studies reveals that nursing interventions based on Watson’s Theory of Human Caring demonstrate consistent and positive effects across various areas of women’s health. These results are grouped under the main themes of reduction in psychological distress, enhancement of self-efficacy and adjustment, and improvement in quality of life and physical well-being.

#### 3.7.1. Reduction in Psychological Distress (Anxiety, Stress, and Depression)

The most prominent finding across the studies is the significant impact of Watson model-based care on reducing anxiety, stress, and depression. Interventions based on Watson’s model significantly decreased depression, anxiety, and stress levels in women following medical abortion [20]. Similarly, caritas-based nursing care improved the mental health of pregnant women who had experienced a previous pregnancy loss, specifically by reducing anxiety and prenatal stress [29]. Positive results were also observed in women facing infertility and cancer. A nursing care program based on THC effectively reduced anxiety and distress in women when infertility treatment failed [25]. In the field of oncology, Watson model-based interventions—including those integrated with reflexology—led to a significant decrease in anxiety and depression scores among women diagnosed with gynecological cancers [9,30]. Furthermore, psycho-educational interventions based on the Watson model were effective in preventing postpartum depression and reducing antenatal anxiety [27,28].

#### 3.7.2. Enhancement of Self-Efficacy, Adjustment, and Coping

Several studies emphasized the role of Watson’s theory in strengthening women’s internal resources and adaptation. Nursing care based on THC significantly increased perceived self-efficacy and adjustment levels in infertile women [24]. In the context of maternal health, a breastfeeding education program grounded in Human Caring Theory significantly improved mothers’ breastfeeding self-efficacy and increased exclusive breastfeeding rates [10]. These findings were further supported by evidence noting that caritas-based postpartum care enhanced both breastfeeding success and the mother’s perception of social support [28]. Additionally, interventions based on this theory improved the active coping strategies of women following unsuccessful IVF cycles [25].

#### 3.7.3. Improvement in Quality of Life and Physical Well-Being

The interventions were also found to be effective in improving overall quality of life and managing physical symptoms. A significant improvement was demonstrated in the global health status and functional scales of the quality of life in patients with gynecological cancer [9]. This was reinforced in a pilot study noting that holistic care based on Watson’s theory helped women manage the physical and psychosocial challenges of cancer more effectively [30]. Furthermore, while the primary focus was psychological, the integration of THC with clinical care was observed to have a positive impact on the overall physical comfort and satisfaction of women in the postpartum and post-abortion periods [10,20].

## 4. Discussion

This systematic review synthesizes current evidence examining the impact of nursing interventions based on Watson’s Theory of Human Caring on women’s mental health. The results from the 10 included studies (9 RCTs, 1 quasi-experimental), involving a total of 869 participants, demonstrate that THC-based approaches are effective in reducing psychological stress, enhancing self-efficacy, and improving quality of life.

The most striking finding of this systematic review is the consistent success of interventions based on Watson’s Theory of Human Caring in reducing anxiety, depression, and stress levels across various clinical stages of women’s health. Synthesized evidence demonstrates that Caritas-based nursing care plays a critical role in supporting mental well-being for women navigating traumatic processes, such as medical abortion, recurrent pregnancy loss, and unsuccessful infertility treatments. Notably, THC-based psychoeducation not only alleviates existing symptoms but also serves as a proactive protective strategy in preventing postpartum depression [27,28]. In the field of oncology, the integration of the theory with holistic methods, such as reflexology, has similarly led to a significant decrease in anxiety and depression scores among patients with gynecological cancer.

Expanding beyond women’s health, the broad utility of THC is evidenced in diverse clinical samples; for instance, an environment and care organized according to THC principles significantly decreased psychological distress in patients undergoing open-heart surgery [33]. Similarly, within pediatric care, implementing Watson’s framework has been synthesized as a crucial instrument for enhancing family-centered care, reducing clinical anxiety, and fostering holistic healing structures for children hospitalized in specialized units [7]. Furthermore, the reach of the theory extends to the professional development of care providers, where clinical education programs grounded in Watson’s theory significantly improve coping strategies and reduce anxiety levels among nursing students [25]. Collectively, these results suggest that the transpersonal caring approach of THC fills the gaps in traditional care models by focusing on the spiritual and existential needs of the patient, thereby providing a robust clinical guide for protecting mental health across various populations.

Another significant finding of this review is that Watson’s Theory of Human Caring extends beyond mere symptom management to strengthen internal resources, specifically by enhancing self-efficacy and adaptive coping mechanisms. In the challenging context of infertility, for instance, THC-based interventions significantly bolster self-efficacy and adjustment levels [24,25]. By fostering a transpersonal caring relationship, the theory enables women to transition from passive distress to active coping strategies. This empowering effect is equally evident in maternal health; THC-based support directly increases breastfeeding self-efficacy, illustrating the practical success of the “transpersonal teaching–learning” process during critical life transitions [10].

Beyond routine clinical adjustments, the profound impact of THC on coping is further highlighted in acute psychological crises. Evidence indicates that caritas-based nursing care can mitigate suicidal behaviors and self-harm by promoting self-reflection, impulse control, and family resilience [34]. Interestingly, this framework’s benefits extend from patients to practitioners; research on nursing students indicates that clinical education programs grounded in Watson’s theory significantly improve stress-coping approaches—specifically by increasing self-confident and social-support seeking behaviors while reducing submissive and unconfident stances [35]. Collectively, these findings suggest that THC serves as a vital framework for promoting resilience, allowing both patients and practitioners to navigate complex health transitions with greater clinical and personal competence.

A further dimension of the findings concerns the impact of Watson’s Theory of Human Caring on quality of life and symptomatic relief in oncological care, where it is often integrated with holistic and physiological interventions. Supporting this approach in thoracic oncology, combining Watson’s theory with the Active Cycle of Breathing Technique (ACBT) not only improved pulmonary function and physical recovery but also significantly lowered anxiety and depression scores in lung cancer patients post-surgery [36]. Similarly, when THC is integrated with complementary methods such as reflexology or aromatherapy, it significantly enhances the quality of life and reduces the symptom burden among women with gynecological cancers [9,30]. Further reinforcing these results, a recent study demonstrated that education based on Watson’s theory effectively reduces depressive symptoms and pain intensity while significantly increasing levels of hope in cancer patients undergoing chemotherapy [37].

Beyond these clinical outcomes, Watson’s theory serves as a vital guide in meeting the expectations for a ‘healing care environment.’ In pediatric oncology, caritas-based care addresses children’s needs for humanistic and compassionate support, facilitating their healthy adjustment to the hospital environment [38]. These collective results suggest that the caritas processes facilitate a synergistic effect, addressing both the physiological discomfort of treatment and the psychological distress of a cancer diagnosis. Ultimately, the theory’s existential dimension proves vital in life-threatening conditions; by helping patients rediscover “meaning and hope,” THC-based care supports spiritual healing and helps patients maintain their dignity and inner strength despite the challenges of a malignant disease.

While these findings strongly highlight the clinical and existential value of humanistic caring, it is prominent to mention that most of the reviewed evidence originates from Turkish studies, alongside one study from Iran. This concentration reflects a well-established and growing trend in the regional literature; over the past few years, Turkish nursing scholars have extensively utilized and implemented Watson’s Human Caring Theory across various clinical fields, ranging from pediatric care and burn management to breastfeeding education and gynecological oncology [8,9,10]. Consequently, while these nursing interventions primarily operate downstream of the broader structural determinants of women’s health [39], as noted in the introduction, macro-level factors such as economic inequalities, rigid gender roles, and heavy caregiving burdens deeply shape women’s underlying vulnerability to mental distress [40]. The interventions reviewed in this study—many of which rely on educational materials, distance learning, or digital platforms like WhatsApp—focus on addressing coping mechanisms at an individual level during intense clinical transitions. Consequently, the actual effectiveness of these caritas-based frameworks may inherently fluctuate depending on a woman’s socioeconomic status, social class, formal education level, and digital resource accessibility [41]. While THC-based protocols provide indispensable humanistic support, they cannot dismantle the upstream structural inequities causing the initial distress. Therefore, future nursing frameworks must integrate equity-sensitive designs, ensuring that digital and educational caring interventions look upstream to accommodate patients whose structural constraints might otherwise limit their engagement with holistic care models [41].

In conclusion, this review demonstrates that Watson’s Theory of Human Caring is a powerful clinical framework that effectively humanizes nursing practice. By addressing the spiritual and existential dimensions of care, THC-based interventions consistently alleviate anxiety and depression while bolstering self-efficacy and hope across diverse clinical settings. Whether managing traumatic health transitions or chronic illnesses, the Caritas processes provide a synergistic effect that transcends traditional biophysical care. Ultimately, integrating Watson’s theory into clinical practice and education serves as a robust strategy for protecting mental health and preserving patient dignity during the most challenging life events.

Despite the significant insights provided, this systematic review has several limitations. Firstly, most of the included studies were conducted in a single geographic region (predominantly Türkiye), which may limit the generalizability of the findings to different cultural and healthcare contexts. Secondly, the variation in the duration, frequency, and specific caritas processes utilized across the interventions introduced a degree of heterogeneity, making it challenging to determine a standardized “dosage” for THC-based care. Thirdly, many studies relied on self-reported scales to measure psychological outcomes, which are subject to subjective bias. Finally, the lack of long-term follow-up in most trials limits our understanding of the sustained impact of these interventions over time. Future research involving multi-center trials with larger, more diverse populations and longitudinal designs is necessary to validate these findings globally.

Another notable limitation of this systematic review relates to the potential issues of equity and intervention accessibility. Several of the included studies relied heavily on psychoeducational materials, distance learning modules, and WhatsApp-based support systems. However, the primary studies did not evaluate whether these digital and educational formats were equally accessible or effective for women with lower formal education levels, limited digital literacy, or restricted technological access. Consequently, our findings may inadvertently overrepresent populations with stable digital resources and higher literacy, masking potential disparities in intervention efficacy among structurally disadvantaged groups.

## 5. Conclusions

This systematic review provides strong evidence that nursing interventions based on Watson’s Theory of Human Caring significantly improve women’s mental health and overall well-being. By prioritizing the transpersonal caring relationship and the caritas processes, these interventions effectively reduce anxiety, depression, and stress while simultaneously fostering hope, self-efficacy, and adaptive coping mechanisms across various traumatic and chronic health conditions. The findings underscore that THC-based care transcends traditional biophysical models by addressing the spiritual and existential needs of the individual, proving particularly effective in oncological care, pregnancy transitions, and clinical education. To ensure the sustainability of these outcomes, it is recommended that THC-based frameworks be integrated into clinical nursing protocols, hospital care environments, and nursing curricula while explicitly accounting for socioeconomic variables. Future clinical models and research designs must look upstream, adopting equity-sensitive approaches to ensure that digital and educational caring interventions are equally accessible and effective for women from disadvantaged backgrounds. Furthermore, subsequent studies should focus on the long-term effects of these interventions and explore their applicability across different global and culturally diverse contexts to validate these findings on a broader scale.

## Figures and Tables

**Table 1:** The list of studies included in the systematic review.

Author(s), Year, Country	Study Design, Year	Objective	Data Collection Tools	Sample Size, Groups and Participant Characteristics	Characteristics of the Intervention	Main Outcomes
Aktürk & Erci, 2019, Türkiye [20]	RCT, 2016–2017	To determine the effect of care given according to Watson’s model on depression, stress, and anxiety (DAS) levels of women who underwent medical abortion	Introductory questionnaire, DAS-42 Scale	I: 52, C: 53, women aged 18+ who had medical abortions between the 10th–20th gestational weeks	Home visits based on Watson’s Model and 10 caritas processes (3 sessions total, weekly); 60–80 min sessions including a care guide training	DAS scores in the experimental group significantly decreased compared to the pretest
Arslan-Özkan et al., 2014, Türkiye [24]	RCT, 2010–2011	To investigate the effects of nursing care based on Watson’s Theory of Human Caring on distress, self-efficacy, and adjustment levels in infertile women	Infertility Distress Scale, Turkish-Infertility Self-Efficacy Scale Short Form, Turkish-Fertility Adjustment Scale	I: 52 C: 53, primary infertility, aged 18–45	4 transpersonal interviews based on Watson’s theory alongside routine care (45–90 min); used caritas processes (4, 5, 6, 7), relaxation exercises, and massage	Significant reduction in infertility distress; self-efficacy and adjustment levels significantly increased compared to the control group
Durgun Ozan & Okumuş, 2017, Türkiye [25]	Single-blind RCT, 2012	To evaluate the effectiveness of the nursing care program based on Watson’s theory on anxiety, distress, and coping when infertility treatment fails	Spielberger State/Trait Anxiety Inventory, Infertility Distress Scale, Ways of Coping Questionnaire	I: 32, C: 35 primary infertility, aged 18+	9-session program based on 10 Caritas factors (treatment phase + after failure); 20–40 min sessions, therapeutic environment, and transpersonal teaching–learning	Anxiety and distress significantly decreased; positive coping styles increased in the intervention group
Durmazoğlu et al., 2024, Türkiye [10]	RCT, 2019–2021	To examine the effect of education/support based on Human Caring Theory on breastfeeding self-efficacy and type of breastfeeding	Baby Feeding Assessment Form, Breastfeeding Self-Efficacy Scale-Short Form	I: 50, C: 50, primiparous, at 28–34 weeks of gestation	12 h of HCT-based sessions, asynchronous distance education modules, and phone support up to 6 months; used caritas processes and visual/tactile materials	Breastfeeding self-efficacy, exclusive breastfeeding rate, and duration were significantly higher in the intervention group
Göral Türkcü & Özkan, 2021, Türkiye [9]	Single-blind RCT, 2016–2018	To examine the effects of reflexology based on Watson’s theory on anxiety, depression, and quality of life in gynecological cancer patients	Beck Anxiety and Depression Inventories, EORTC QLQ-C30 v3.0	I: 31, C: 31, Stage II–III gynecological cancer diagnosis, receiving chemotherapy	Reflexology applied 3 times a week (total 6 sessions, 30–45 min) based on the 6th caritas factor (creative problem solving)	Anxiety and depression decreased; quality of life increased; symptoms like pain, fatigue, and loss of appetite significantly reduced.
Maryam et al., 2010, Iran [26]	Quasi-experimental study, 9 weeks	To investigate the effect of an exercise program on the quality of life (QOL) in women with breast cancer receiving chemotherapy	Demographic and clinical form, QOL-Breast Cancer (QOL-BC) questionnaire	I: 28, C: 28, breast cancer stages 0–3, receiving chemotherapy	Home exercises for 20–30 min, 3–5 days/week for 9 weeks (warm-up, main training, cool-down); used music and visual CDs	Significant improvement in physical, emotional, social, and total QOL scores in the experiment group.
Özhüner & Özerdoğan, 2024, Türkiye [27]	RCT, 2022–2023	To evaluate the impact of a Watson model-based psycho-educational intervention on preventing PPD and social support perception	Edinburgh Postpartum Depression Scale (EPDS), Multidimensional Scale of Perceived Social Support (MSPSS)	I: 45, C: 46, at 20–32 weeks of gestation	6-session psychoeducation (4 antenatal, 2 postnatal); used education booklet, homework, and relaxation exercises	EPDS scores significantly decreased; perception of friend and family support significantly increased compared to the control group
Özhüner & Özerdoğan, 2025, Türkiye [28]	RCT, 2024–2025	To evaluate the effect of psychoeducational intervention on PPD and breastfeeding self-efficacy outcomes	EPDS, Antenatal/Postpartum Breastfeeding Self-Efficacy Scales	I: 50, C: 52, at 20–34 weeks of gestation	6-session psychoeducation based on Watson’s Human Caring Model (4 pregnancy, 2 postnatal); practical breastfeeding education and emotional support	Postpartum breastfeeding self-efficacy increased and the risk of PPD significantly decreased in the intervention group.
Tektaş & Çam, 2017, Türkiye [29]	RCT, 2013–2014	To determine the effects of care based on Watson’s Theory on the mental health of pregnant women after a loss	Beck Anxiety, Depression, and Hopelessness Scales, Prenatal Attachment Inventory	I: 55, C: 46, history of pregnancy loss, pregnant <12 weeks	5 individual interviews (weeks 10–28) based on 10 healing processes; used therapeutic touch, music, and relaxation exercises	Anxiety, depression, and hopelessness scores significantly decreased; prenatal attachment levels significantly increased.
Teskereci et al., 2022, Türkiye [30]	Single-blind RCT, 2014–2016	To determine the effects of a Watson model-based program on chemotherapy symptoms, hope, and meaning in life	CSAS, Herth Hope Scale, Life Attitude Profile (LAP)	I: 26, C:26, Stage II–III gynecologic cancer, receiving chemotherapy	5-session individual program (~90 min) based on 10 Caritas processes; used aromatherapy, music, imagination, and a care guide	Significant decrease in some chemotherapy symptoms; significant increase in hope and meaning in life scores.

Abbreviations: CSAS: Chemotherapy Symptom Assessment Scale; DAS-42: Depression, Anxiety, and Stress Scale; EORTC QLQ-C30: European Organisation for Research and Treatment of Cancer Quality of Life Questionnaire; EPDS: Edinburgh Postpartum Depression Scale; HCT: Human Caring Theory; I/C: Intervention Group/Control Group; LAP: Life Attitude Profile; MSPSS: Multidimensional Scale of Perceived Social Support; PPD: Postpartum Depression; QOL/QOL-BC: Quality of Life/Quality of Life-Breast Cancer; RCT: Randomized Controlled Trial.

**Table 2:** Quality assessment scores of the studies.

Studies	JBI Critical Appraisal Checklist Questions for Randomized Controlled Studies													Quality Score of the Study
	Q1	Q2	Q3	Q4	Q5	Q6	Q7	Q8	Q9	Q10	Q11	Q12	Q13	
Aktürk & Erci, 2019 [20]	Y	N/A	Y	N	N	N	Y	Y	Y	Y	Y	Y	Y	69.2%
Arslan-Özkan et al., 2014 [24]	Y	Y	Y	N	N	N	Y	Y	Y	Y	Y	Y	Y	76.9%
Durgun Ozan & Okumuş, 2017 [25]	Y	N/A	Y	N	N	N	Y	Y	Y	Y	Y	Y	Y	69.2%
Durmazoğlu et al., 2024 [10]	Y	N/A	Y	N	N	N	Y	Y	Y	Y	Y	Y	Y	69.2%
Göral Türkcü & Özkan, 2021 [9]	Y	N/A	Y	N	N	Y	Y	Y	N	Y	Y	Y	Y	76.9%
Özhüner & Özerdoğan, 2024 [27]	Y	N/A	Y	N	N	Y	Y	Y	Y	Y	Y	Y	Y	69.2%
Özhüner & Özerdoğan, 2025 [28]	Y	N/A	Y	N	N	N	Y	Y	Y	Y	Y	Y	Y	69.2%
Tektaş & Çam, 2017 [29]	Y	N/A	Y	N	N	N	Y	Y	Y	Y	Y	Y	Y	69.2%
Teskereci et al., 2022 [30]	Y	N/A	Y	N	N	Y	Y	Y	Y	Y	Y	Y	Y	76.9%
Question quality score	100%	10%	100%	0%	0%	0%	100%	100%	%90	100%	100%	100%	100%	
	JBI Critical Appraisal Checklist Questions for Quasi-experimental Studies													
Maryam et al., 2010 [26]	Y	Y	Y	Y	Y	Y	Y	Y	Y					100%
Question quality score	100%	100%	100%	100%	100%	100%	100%	100%	100%					

Abbreviations: Y: Yes; N: No; N/A: Not Applicable.

## Data Availability

No new data were created or analyzed in this study. Data sharing is not applicable to this article.
